# Occurrence and applications of CRISPR-Cas systems in bifidobacteria

**DOI:** 10.1128/aem.01703-25

**Published:** 2026-01-05

**Authors:** Jiyong Shin, Rodolphe Barrangou

**Affiliations:** 1 Department of Food, Bioprocessing and Nutrition Sciences, North Carolina State University 6798 , Raleigh, North Carolina, USA; The Pennsylvania State University, University Park, Pennsylvania, USA

**Keywords:** *Bifidobacterium*, genome editing, Cas, CRISPR

## Abstract

*Bifidobacterium* is a key member of the human gut microbiota, and many strains are widely used as probiotics due to their health-promoting properties. Despite growing interest, genetic studies in *Bifidobacterium* have been relatively limited, primarily due to the lack of available genome editing tools. Recent advances in genomics and CRISPR-Cas systems provide opportunities for targeted genome modification in this genus. In this review, we provide an overview of the occurrence, diversity, and distribution of CRISPR-Cas systems across *Bifidobacterium* species and examine the editing tools developed and implemented to date. We also highlight practical challenges such as strain variability and low transformation efficiency and introduce future avenues of research such as large-payload insertion and *in situ* editing. Expanding the genetic toolbox for *Bifidobacterium* will broaden our understanding of this important genus and enable the development of next-generation probiotics.

## INTRODUCTION

Bifidobacteria are among the dominant early colonizers in the human gut and are suggested to be drivers of host health (1). Over the past decades, studies have highlighted various roles of bifidobacteria, such as modulation of the host immune response, establishment and maintenance of gut microbiome stability through synergistic interactions, prevention of pathogen proliferation, and prevention of irritable bowel syndrome and ulcerative colitis (2–5). Moreover, bifidobacteria are also known to produce important metabolites, including multiple vitamins, short-chain fatty acids, and bacteriocins (6–8). Due to these beneficial effects, bifidobacteria have been widely formulated in the food supply chain, including beverages, fermented dairy products, and dietary supplements (9). As a matter of fact, bifidobacteria are only second to lactobacilli in overall commercial probiotic product composition and are sometimes the dominant genus in probiotic products (10).

Although the positive health benefits of *Bifidobacterium* have been supported by accumulating literature evidence, there has been relatively limited research on its molecular mechanisms of action and on identifying the genetic basis for select health-promoting functions. However, with advances in sequencing technologies, more extensive genomic analyses have been carried out to explore its genomic potential, metabolic pathway repertoire, and gene functions (11–13). Still, functional studies have been relatively limited, mostly due to the lack of available genome editing tools and the recalcitrant nature of *Bifidobacterium*, making it difficult to manipulate. To address this, different genome editing strategies have been recently developed and applied in some model species and reference strains.

Most of the early methods of bifidobacterial mutagenesis strategies involved the use of non-replicative plasmid insertion. Single and double crossover insertion methods involve integration of the whole plasmid or an insertion marker into the gene of interest through homologous recombination (14, 15). Later, marker-less strategies were developed to remove the selection marker from the genome, generating a clean deletion (16). However, this method requires extensive screening after the second recombination due to the inability to select between the wild type and the marker-less mutant. Alternatively, gene functions have also been studied by generating mutant libraries using a transposon mutagenesis system (17). This method enables rapid high-throughput screening of gene function but does not enable a predictable genotype of the mutant, since it does not rely on precise editing.

Clustered regularly interspaced short palindromic repeats (CRISPR) and associated Cas proteins constitute the adaptive immune system in prokaryotes, which provides defenses against invasive nucleic acids (18). This system operates through three steps involving adaptation, expression, and interference (19) in a DNA-encoded (18), RNA-guided (20), nucleic acid-targeting manner (21, 22). In the adaptation step, invasive sequences are integrated into the CRISPR array as spacers by the Cas1 and Cas2 proteins (23). Subsequently, in the expression and phases, these spacers are transcribed and processed into CRISPR RNAs (crRNAs) that assemble with Cas nucleases. During interference, the crRNA-Cas complex recognizes the complementary target sequence and cleaves the nucleic acid (24). By designing guide sequences to direct the Cas effector protein, this mechanism can be repurposed as a programmable genome editing tool (25).

While genome editing in eukaryotes has progressed rapidly through the characterization and use of single-effector proteins such as Cas9 and Cas12, the development of prokaryotic editing tools has been relatively slow and limited in scope and adoption (26). However, unlike in eukaryotes, the abundance of CRISPR-Cas systems in bacteria, specifically in bifidobacteria, offers opportunities to utilize both exogenous and endogenous Cas effectors (27). The exogenous CRISPR-Cas system can be repurposed using a self-targeting guide along with a homologous recombination editing template in the absence of a functional endogenous system (28). Conversely, endogenous CRISPR-Cas systems can also be repurposed using the same general strategy, using only a self-targeting array without the need for an external Cas protein (29–31). Recently, different CRISPR-Cas-based editing methods have been developed for bifidobacteria, which, compared to earlier mutagenesis methods, offer improved precision, efficiency, and specificity and require fewer steps to obtain an edited strain.

In this review, we analyze the occurrence and distribution of CRISPR-Cas systems in a recently expanded genome data set of *Bifidobacterium*. We also examine the current state of CRISPR-based editing tools developed for *Bifidobacterium* and discuss their limitations. Finally, we propose future directions for incorporating CRISPR-Cas systems to enable more versatile and targeted genome editing, illustrating their potential for next-generation probiotic development and the manipulation of the composition and function of microbiomes.

## OCCURRENCE AND DISTRIBUTION OF CRISPR-CAS SYSTEMS IN *BIFIDOBACTERIUM*

The continued development of sequencing technologies over the past decades has led to a wealth of genome sequences and the genesis of comprehensive bacterial genome databases. Concurrently, advances in computational biology and bioinformatics have enabled the analysis and deciphering of diverse bacterial genomes. Of note, several CRISPR-centric pipelines and computational tools have emerged to determine the occurrence, distribution, and composition of CRISPR-Cas systems in bacteria (32–34). To date, CRISPR-Cas systems are classified into 2 major classes, 7 types, and 46 different subtypes based on the structure and function of the Cas proteins (35). Previous studies analyzing the occurrence of CRISPR-Cas systems in *Bifidobacterium* used 48 genomes in 2015, which increased to 954 in 2020 (27, 36). In this study, it was further expanded (over 100× in a decade), and a total number of 6,657 *Bifidobacterium* genomes available through NCBI, as of February 2025, were analyzed using CRISPRCasTyper version 1.8.0, which identifies systems based on CRISPR arrays and *cas* operon organization (33). Previous analysis of *Bifidobacterium* and performance test of CRISPRCasTyper have shown that Type II-C systems are relatively harder to detect (27, 33, 36). Therefore, in this study, loci predicted as Type II-Cs based only on the CRISPR arrays were manually checked for the proper corresponding *cas* operon in this analysis.

**Fig 1:**
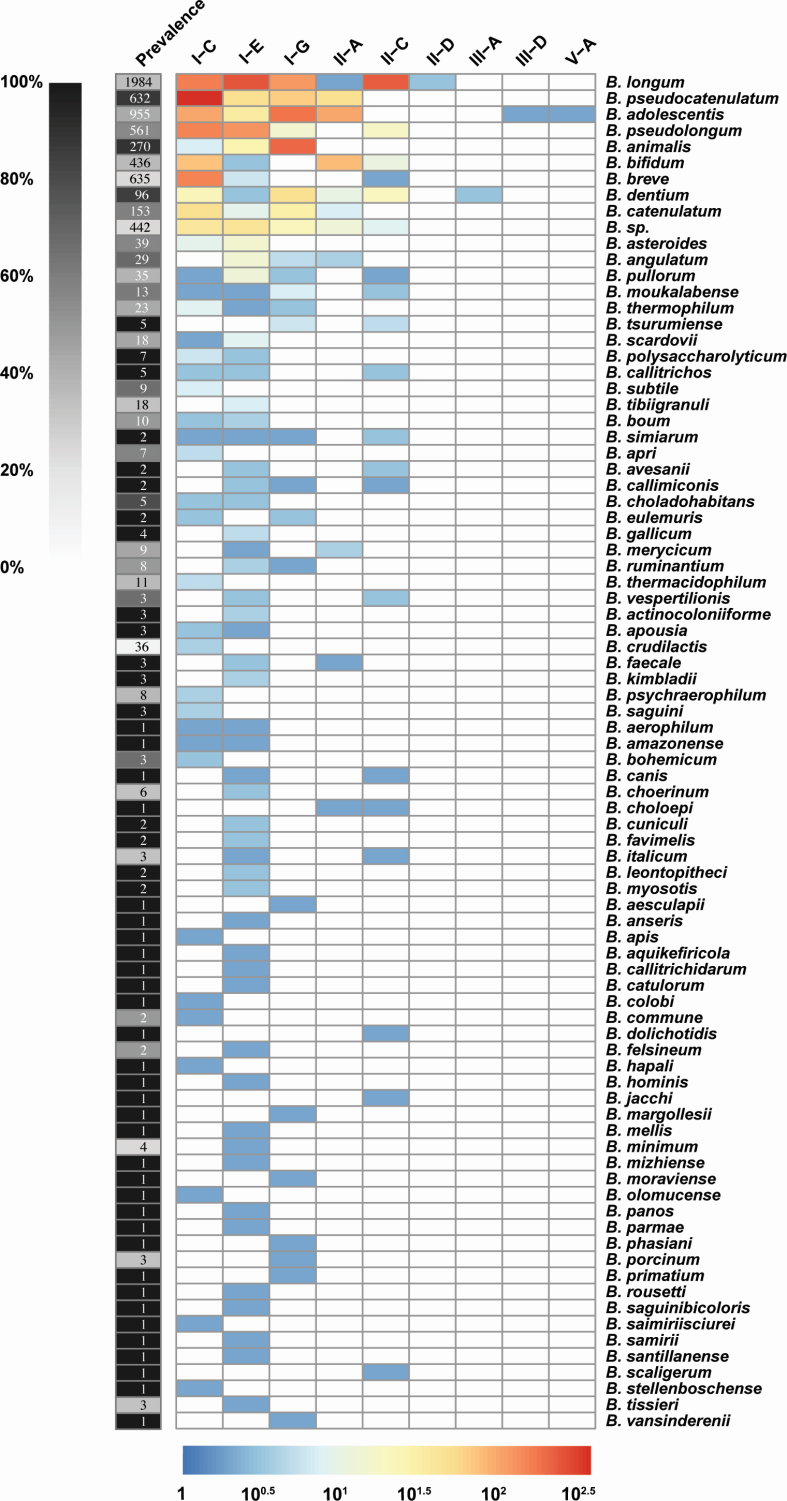
Distribution of CRISPR-Cas subtypes in the *Bifidobacterium* genus that encode a CRISPR-Cas system. The left heatmap shows the number of genomes analyzed and the prevalence (percentage) of CRISPR-Cas systems per species. The right heatmap shows the number and distribution of each subtype at the species level on a log scale. The heatmaps were plotted using the pheatmap package (1.0.12) (37). The 27 species without a CRISPR-Cas system and not shown in are *B. aemilianum*, *B. apicola*, *B. aquikefiri*, *B. biavatii*, *B. bombi*, *B. castoris*, *B. cebidarum*, *B. coryneforme*, *B. criceti*, *B. erythrocebi*, *B. fermentum*, *B. goeldii*, *B. imperatoris*, *B. indicum*, *B. lemurum*, *B. magnum*, *B. miconis*, *B. miconisargentati*, *B. mongoliense*, *B. oedipodis*, *B. platyrrhinorum*, *B. pluvialisilvae*, *B. pongonis*, *B. ramosum*, *B. reuteri, B. simiiventris*, and *B. xylocopae*.

Overall, of the 6,657 strains analyzed, 3,050 genomes (45.8%) encoded a CRISPR-Cas system, with 133 encoding multiple systems (Table 1 and Fig. 1). These CRISPR-Cas systems were distributed across 83 out of 110 species (75.5%), which had at least one CRISPR-Cas system. Among them, *Bifidobacterium longum* had the largest number of strains (714), which encode a CRISPR-Cas system showing an occurrence rate of 36%. Within species with more than 10 strains analyzed, *Bifidobacterium animalis* showed the highest occurrence rate of 89.3%. Overall, and consistent with general trends across bacteria, Type I was the most dominant CRISPR-Cas type (85.3%), with the Type I-C system present in the highest number of strains, while Type I-E was found across the greatest number of species. Although the number of analyzed genomes has increased significantly, there is still variability and bias in the distribution of CRISPR-Cas systems across strains and species observed, mainly due to the uneven and hypervariable number of genomes available for each species.

**TABLE 1:** Number of species and strains of the *Bifidobacterium* genus with CRISPR-Cas systems for each system subtype

	Number of strains	Number of species
Total	6,657	110
With CRISPR-Cas system	3,050	83
Without CRISPR-Cas system	3,607	27
I-C	1,212	37
I-E	663	56
I-G	728	23
II-A	262	10
II-C	315	19
II-D	2	1
III-A	2	1
III-D	1	1
V-A	1	1

Compared to the most recent study on the comprehensive mining of CRISPR-Cas systems in *Bifidobacterium* (27), the new and expanded analysis was able to identify 35 new species with CRISPR-Cas systems. In addition, we were able to identify new subtypes, Types II-D, III-A, III-D, and V-A, in *Bifidobacterium*. Specifically, Type II-D was encoded in *B. longum,* and Type III-A was encoded in *B. dentium*. Types III-D and V-A were both encoded in *B. adolescentis*.

Overall, the occurrence of CRISPR-Cas systems was not strictly species-dependent; however, certain species with a large number of sequenced genomes did show dominance of specific subtypes (Fig. 1). For example, Type I-G was dominant in *B. animalis*, while Type I-C was dominant in *B. breve*. The broad distribution of Type I systems across *Bifidobacterium* suggests that they are the most established and likely the best candidates for repurposing in endogenous CRISPR-Cas-based genome editing. Nonetheless, the diversity in subtype distribution also shows that it is not feasible to develop a single, universally applicable CRISPR-Cas system for all species, let alone strains. Rather, the editing approach will depend on the specific species-strain combination of interest. Nevertheless, knowing which systems are most common provides a valuable foundation for selecting a CRISPR-Cas type that would be broadly applicable across the genus, species, and strains of interest.

## CRISPR-BASED GENOME EDITING TOOLS IN *BIFIDOBACTERIUM*

**Fig 2:**
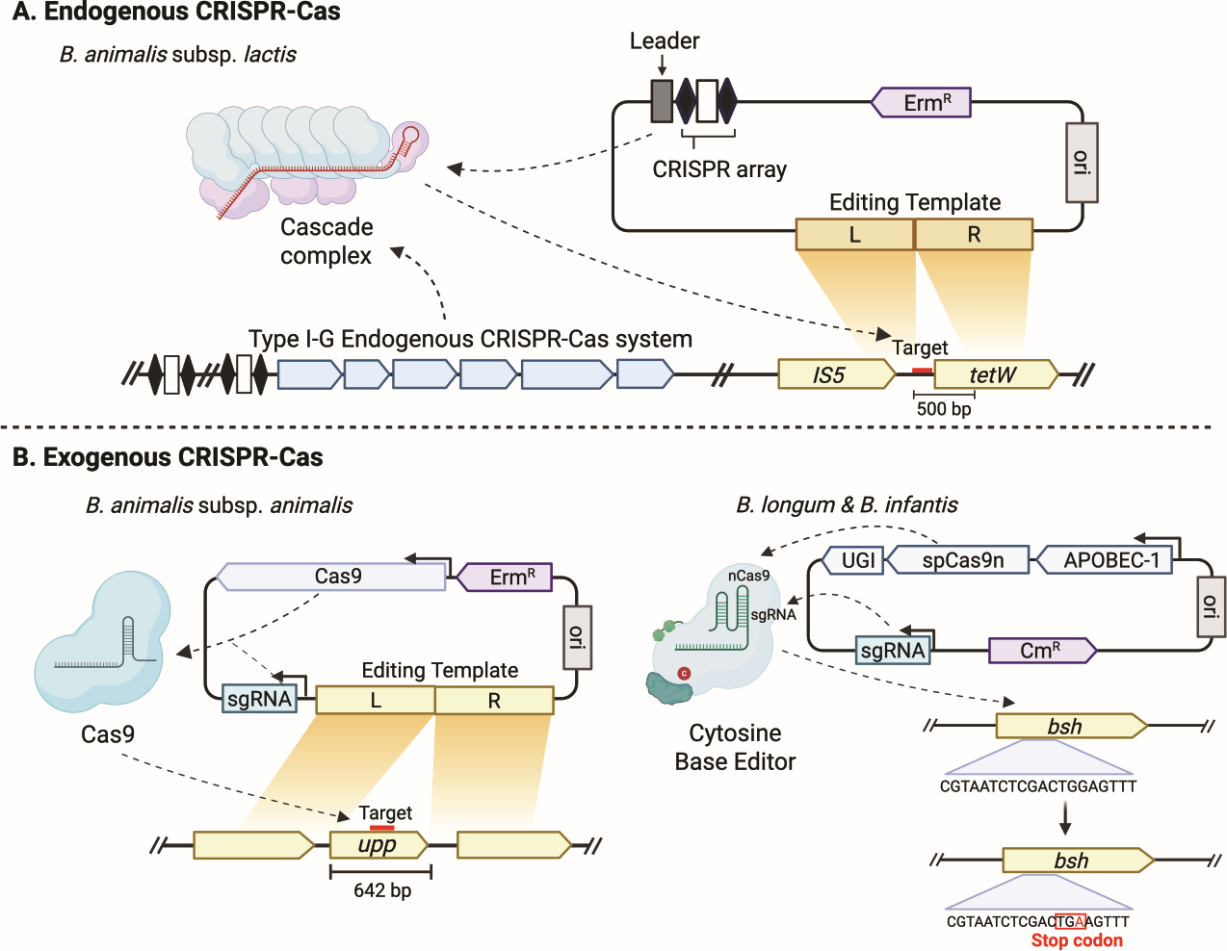
CRISPR-based editing tools used in *Bifidobacterium*. (**A**) Endogenous CRISPR-based editing: Type I-G system repurposed to generate a 500-bp deletion in the *tetW* gene of *B. lactis* (47). (**B**) Exogenous CRISPR-based editing: (left) Cas9 protein introduced to delete the *upp* gene in *B. animalis* subsp. *animalis* (45). (Right) Cytosine base editor applied in *B. longum* subsp. *longum* and *B. longum* subsp. *infantis* to introduce a premature stop codon in the *bsh* gene (46).

CRISPR-Cas-based genome editing tools have been developed rapidly over the past decade in various organisms. In lactic acid bacteria, tools generating deletion, insertion, and single base alteration have been applied to enable the study and manipulation of lactic acid bacterial species (38). However, due to the recalcitrant characteristics of *Bifidobacterium*, most of the research has focused on other lactic acid bacteria, primarily *Lactobacillus* (39). In these species, a wide range of exogenous CRISPR-Cas systems, notably Cas9, nCas9, dCas9, base editors, and CAST/CasTn (CRISPR-associated transposons), have been utilized (30, 40–43). Additionally, various endogenous CRISPR-Cas systems, such as Types I-C, I-G, I-E, and II-A, have been explored and used for editing. On the other hand, only a limited number of studies have successfully edited *Bifidobacterium* using CRISPR-Cas systems (44–47) (Fig. 2).

Although there may be differences in the design and usage of endogenous and exogenous CRISPR-Cas systems, the overall editing mechanism remains the same. The guide RNA directs the Cas effector to the complementary target site in the genome, introducing DNA damage. An editing template designed upstream and downstream of the target site facilitates homologous recombination, resulting in the intended edit. However, it remains unclear whether homologous recombination systematically follows the double-stranded break (DSB) showing homology-directed repair, or whether homologous recombination itself introduces the intended edit while the CRISPR-Cas system primarily serves to counter-select against the wild-type genotype. A recent study has shown enhanced editing efficiency by inducing the expression of Cas protein in bacteria, suggesting that the mechanism relies more on counterselection rather than homology-directed repair (48).

### Endogenous CRISPR-based editing

Considering the wide distribution of CRISPR-Cas systems in *Bifidobacterium*, harnessing the endogenous CRISPR-Cas system has practical advantages over introducing exogenous Cas effectors (Fig. 2A). Introducing portable Cas effectors can be difficult due to their intrinsic cytotoxicity (49). Additionally, as it only requires a mini CRISPR array encompassing a self-targeting spacer complementary to the gene of interest and an editing template, its smaller size enables enhanced delivery of the plasmid (47, 50, 51).

Due to these advantages, the first CRISPR-Cas-based editing was conducted by characterizing and repurposing the endogenous Type I-G CRISPR-Cas system in *B. animalis* subsp. *lactis* (*B. lactis*) (47). Using a self-targeting CRISPR array without an editing template, the study screened for large, naturally occurring variants with deletions of up to 27 kb. With the introduction of a 1.2-kb editing template, a 500-bp deletion was successfully generated in the *tetW* gene, eliminating the tetracycline resistance and restoring antibiotic sensitivity. Subsequently, a study was able to delete the whole coding sequence of the uracil phosphoribosyl-transferase gene (993 bp) by repurposing the endogenous Type I-C CRISPR-Cas system in *B. breve* (44). Additionally, by altering the design of the editing template, the study demonstrated other means through which the gene could be inactivated. A stop codon was introduced through single base substitution and insertion of three consecutive stop codons, resulting in the successful inactivation of the *upp* gene.

### Exogenous CRISPR-based editing

Despite the significant advantages of repurposing endogenous CRISPR-Cas systems, according to our analysis, over half of the genomes do not encode a CRISPR-Cas system. A feasible strategy in this case would be to introduce an exogenous CRISPR-Cas system (Fig. 2B). Although Class I CRISPR Cas systems are the most prevalent and generally exhibit less toxicity, they require multiprotein complexes such as the CRISPR-associated complex for antiviral defense (Cascade) for cleavage, making their engineering delivery more challenging. Class 2 systems, on the other hand, encode single subunit effectors for cleavage and are therefore more commonly used exogenously (35).

The commonly used signature proteins for DNA editing are Cas9 and Cas12 for type II and type V systems, respectively. As mentioned earlier, in endogenous CRISPR-Cas editing, a mini CRISPR array can be introduced through the plasmid as it is processed into mature crRNA through the native CRISPR-Cas system. Similarly, Cas12 can independently process its own crRNA, which is needed for target recognition and cleavage (52). However, Cas9 alone is not able to process a CRISPR array into crRNA and trans-activating crRNA, as it depends on host factors like RNase III (24). Therefore, a single gRNA must be introduced along with Cas9 to ensure its functionality (25). Most importantly, the introduction of an exogenous CRISPR-Cas system requires a larger plasmid, making transformation more challenging.

To illustrate the potential of the exogenous CRISPR-Cas system, Li et al. (45) were able to utilize Cas9 protein to obtain pure mutant knockouts of genes 0348 (972 bp) and 0208 (642 bp), which encode uracil phosphoribosyl transferase. For larger deletions, this study was able to obtain a mixed colony of wild-type and mutant cells with 3,000-bp deletion but was unable to obtain any colony with a pure mutant strain. While the editing tools mentioned above performed plasmid curing through passaging of the cells without the selection marker, this study introduced an inducible plasmid curing system in which they introduced a second gRNA targeting the selection marker and removed the plasmid after the edit was performed.

### Base editing

The two methods mentioned above generate a DSB and require a template with homology arms for homologous recombination. Genome editing methods based on DSBs are more error-prone and may lead to undesired insertion or deletion (53). Additionally, not only does the long editing template reduce the transformation efficiency due to its size, but the length of the editing template also affects the overall editing efficiency, making it difficult to develop a uniform, high-efficiency editing tool. In contrast, base editors have the potential to overcome these challenges by forgoing the need to form a DSB and requiring an editing template (54). Base editors fuse a catalytically impaired CRISPR-Cas9 mutant (dCas9 or nCas9) to a DNA deaminase (55). The CRISPR-Cas9 mutant directs the base editor to the target site through the guide RNA spacer, and the deaminase assists in catalyzing A·T-to-G·C conversions. Two types of base editors have been developed based on the deaminases: cytosine base editor (CBE) and adenosine base editor (ABE) (55, 56).

To date, only CBE has been applied in *Bifidobacterium*. Initially, Pan et al. (47) used CBE to introduce a premature stop codon into the *tetW* coding sequence, resulting in the loss of the resistance phenotype. Subsequently, Lin et al. (46) further developed CBE by utilizing a CBE with a UGI linker, which increases the base editing efficiency. Additionally, different promoter combinations were used to increase gRNA expression while reducing Cas9n expression. The developed base editor was able to introduce a premature stop codon in multiple genes in multiple *Bifidobacterium* species, including *B. adolescentis, B. longum* subsp. *longum, B. longum* subsp. *infantis* (*B. infantis*), demonstrating its versatility and broad applicability (Fig. 2B).

Despite these advancements, base editors still have some inherent limitations in *Bifidobacterium*. Off-target effects can occur within the editing window, and when introducing a premature stop codon, only certain codons can be converted into a stop codon, which limits where edits can be made (across and within genes of interest). Another limitation that restricts where edits can be made in the genome is the protospacer adjacent motif (PAM) sequence requirement. Unlike the other limitations mentioned above, this issue can be addressed by engineering the base editor. For example, one study modified different components of the base editor to enhance its application in *Streptomyces* (57). More specifically, SpCas9 was engineered to recognize a more flexible NG PAM instead of NGG, while also reducing off-target effects. The deaminase was also replaced to improve editing efficiency in high-GC content regions. Although this exact system might not work the same way in *Bifidobacterium*, a similar approach could be taken to improve editing efficiency and flexibility.

In addition, ABE could be developed for use in *Bifidobacterium*, allowing researchers to study how single amino acid changes impact gene function. Along with CBE, this would be a powerful tool for making quick edits, since changes can be made by simply modifying the gRNA sequence without the need for an editing template. Expanding and optimizing editing tools, such as base editors, would help broaden the genetic toolbox available for studying and engineering *Bifidobacterium*.

Ultimately, the choice of genome editing tool depends on the strain and target of interest, as each method has its own advantages and drawbacks. Introducing an exogenous Cas effector or a complete base editor can burden the cell due to the intrinsic cytotoxicity of the Cas proteins, negatively affecting the editing efficiency. Nevertheless, reports of CRISPR-based editing in *Bifidobacterium* show a wide range of efficiencies across systems, indicating that multiple factors influence outcomes. Additionally, the expression level of the Cas effector or the crRNA, as well as the length of the editing template, may influence the editing efficiency (58). In addition, chromosomal organization shaped by nucleoid-associated proteins and the epigenetic state influencing gene transcription may also affect the editing outcome (59, 60).

## CHALLENGES AND OPPORTUNITIES FOR GENOME EDITING IN *BIFIDOBACTERIUM*

### Low transformation efficiency

*Bifidobacterium* lacks standardized, validated molecular parts, such as promoters, terminators, and reporters, as well as broadly compatible plasmid backbones, which constrains predictable engineering across species (61). Operationally, the initial and most fundamental step to enable editing in *Bifidobacterium* is to transfer genetic material into the cell. However, this process is challenged by the recalcitrant nature of *Bifidobacterium*, including its refractory cell wall, the abundance of restriction-modification (R-M) systems, and limited molecular tools, plasmid backbones, and transformation protocols, resulting in relatively low transformation efficiency, and tools available only in a limited range of species (62).

The multilayered and complex cell wall of *Bifidobacterium* presents a major barrier for gene delivery, as the most common method of transformation in *Bifidobacterium* is electroporation. The efficiency of electroporation depends on the structure and density of the cell wall (63, 64). Efforts have been made to enhance transformation efficiency by increasing the fragility of the cell wall. For example, in *B. bifidum* BGN4, the addition of 0.2 M NaCl, a cell wall weakening agent, has shown a 20-fold increase in transformation efficiency (65). In addition to the chemical treatment to weaken the cell wall structure, recent work has demonstrated that transformation efficiency is also influenced by the expression level of key competence genes (66). The identified genes are relevant to DNA binding or DNA transmembrane transport, both relevant to the uptake of foreign DNA.

Another significant hurdle in transformation is the abundant R-M systems identified in *Bifidobacterium*. The R-M system functions as a barrier against foreign DNA as it recognizes unmethylated short DNA sequences and cleaves them. The significance of the R-M system on transformation has been demonstrated in *Bifidobacterium longum* subsp. *longum* NCIMB 8809, where knockout of restriction endonuclease genes resulted in a significant increase in transformation efficiency (46). The occurrence and diversity of anti-CRISPR systems may also be a contributing factor (67). Although they have not yet been experimentally validated, these systems have been predicted bioinformatically in bifidobacteria (68).

To circumvent the challenges posed by R-M systems, two primary strategies have been developed. The first strategy is to methylate the plasmid before introducing it into the bacterium. Here, a foreign methylase gene is cloned into *Escherichia coli*, allowing the shuttle vector to be pre-methylated by propagating it in that *E. coli* host. This method was first introduced and applied to *B. adolescentis* (69) and *B. breve* (70) and was later shown to be applicable to *B. bifidum* (71), *B. lactis* (72), and *B. longum* subsp. *longum* (73), all showing improvement in the transformation efficiency. A similar approach was applied by generating a REase mutant *Bifidobacterium* strain and passaging the plasmid to methylate the DNA, allowing it to later bypass the native R-M system in the wild-type strain (46). However, this method requires an additional cloning step, which may result in other complications during plasmid construction. Recently, a cell-free transcription-translation system was utilized to methylate DNA by expressing methyltransferase *in vitro*, offering a cloning-free alternative that enhanced transformation efficiency in *B. breve* and *B. longum* subsp. *longum* (74).

The second strategy is to remove R-M recognition sites by altering the nucleotide sequence to alternative synonymous codons to make an R-M-insensitive vector (75). While this method may seem simple, as it also does not require cloning or methylation steps, the limitation of this approach is that base substitution would not be applicable when the R-M motif is located in the replicon of the plasmids. In this case, we would need to replace the replicon, but considering the limited availability of suitable replicons, base substitution is not always an option (61).

Although less commonly applied, conjugation offers an alternative approach for delivering genetic material with distinct advantages over electroporation. During conjugation, the donor contacts the recipient and mobilizes the plasmid by threading single-stranded DNA through a secretion channel. The recipient then copies the second strand, establishing the plasmid. Therefore, the delivery efficiency is typically not affected by the size of the vector, and it also bypasses the R-M system because methylation occurs after the second-strand synthesis in the recipient (76). A conjugation method was developed for delivering genetic material from *E. coli* to different *Bifidobacterium* subspecies (*B. lactis*, *B. bifidum*, *B. breve*, *B. infantis*, and *B. longum* subsp. *longum*), providing an alternative to electroporation (76). Additionally, a plasmid facilitating conjugation between *B. breve* and *B. longum* subsp. *longum* was identified (77). Notably, conjugation-mediated CRISPR interference (CRISPRi) has been demonstrated in *B. catenulatum*, showing successful plasmid delivery and effective transcriptional repression (78). Although not many attempts have been reported to be successful, its potential to overcome the limitations of electroporation suggests that further optimization could enhance gene transfer efficiency and expand the genetic tools available for *Bifidobacterium* research.

### Strain variability

Despite recent efforts to enhance transformation efficiency by addressing barriers, such as R-M systems and cell wall structure, most of these solutions are strain-specific, with no universal protocol applicable across the *Bifidobacterium* genus. Transformation conditions must be tailored to the specific strain due to the variability at the genomic and epigenomic levels (61).

Building on the approach used in *B. breve*, *B. bifidum* transformation was similarly optimized by identifying strain-specific methylation patterns through REBASE and methylome sequencing (79). Both studies in *B. breve* and *B. bifidum* started with the same plasmid (pNZ44), but due to the removal of target strain-specific R-M motifs, they resulted in two different plasmids (pNZ003 and pNZ123). Moreover, both studies required strain-specific adjustments in the transformation protocols, such as carbohydrates in the growth media, amount of plasmid DNA, electroporation parameters, and recovery media. Ultimately, both studies were able to improve the transformation efficiency by 10^3^-fold compared to the earlier protocol (75, 79, 80).

Supporting this observation, in a study that was developed for broader application across six subspecies, not only was the overall transformation efficiency lower compared to the strain-specific optimized protocol, but it also varied across species, showing up to 100-fold difference between subspecies (81). One of the contributing factors to this strain variability is the methylation pattern. Although a conserved methylation pattern across *Bifidobacterium* species has been identified, the strain-specific differences in epigenetic pattern ultimately affected the transformation efficiency (47).

Taken together, these findings demonstrate that while the contributing factors for low transformation efficiency of *Bifidobacterium* are better understood and can be overcome, the developed plasmids and protocols remain largely strain-specific (65, 80). Moving forward, in addition to the removal of the R-M motifs from plasmids or methylation of the plasmid, parameters in the transformation protocol, including media composition, plasmid amount, and electroporation conditions, will need to be tailored to the strain of interest (61, 66).

### Large payload insertion

CRISPR-Cas-based genome editing in *Bifidobacterium* has enabled gene deletion, base editing, and short insertions of stop codons. However, insertions beyond three stop codons are yet to be established. Insertion of multi-kilobase-long sequences encoding whole metabolic operons, biosynthesis clusters, or even a whole CRISPR-Cas system could open new opportunities for applications in *Bifidobacterium.* This could lead to the development of next-generation probiotics with enhanced and novel functional properties and metabolic pathways.

Although successful large insertions in *Bifidobacterium* have yet to be reported, several studies have demonstrated successful reports of large insertions in other organisms. CRISPR-associated transposases (CASTs/CasTn) are programmable, RNA-guided DNA insertion systems that combine CRISPR-Cas effectors and transposases to mediate site-specific integration, independent of homologous recombination and DSBs (82, 83). Notably, the Type I-F CAST system, which was originally characterized from *Vibrio cholerae,* was adapted for its use in *Lactococcus lactis,* demonstrating insertion of up to 10 kb (43). For CAST to be applicable in *Bifidobacterium*, the major hurdle would be the delivery of both the insertion sequence and the large CAST machinery, along with further optimization for system expression.

Another method for programmable large insertion, independent of homologous recombination and DSBs, hinges on large serine recombinases (LSRs). LSRs are site-specific DNA recombinases that direct integration of mobile genetic elements into the bacterial genome (84). These enzymes catalyze the recombination of two attachment sites, one in the bacterial genome (*attB*) and the other in the mobile genetic element, resulting in unidirectional integration. Similar to CAST, the delivery of large payload genes would be a challenge. What is different for LSRs is that landing pads (*attB*) would first need to be inserted into the genome for the system to be functional. The length of *attB* typically ranges between 35 and 50 bp, and although insertion may be possible through non-replicative plasmid insertion, no studies have yet demonstrated clean insertions of this length without the plasmid backbone in *Bifidobacterium*. Despite these barriers, LSRs remain attractive due to their modularity and stable integration capacity.

Moreover, further optimization of LSRs can be achieved to improve their function in *Bifidobacterium*. Other studies have expanded on LSRs, such as PASSIGE, a system that combines prime editing with LSRs to enable precise insertion of large DNA cargoes over 10 kilobases in mammalian cells (85). Although the system itself is not compatible with bacterial systems, this study used phage-assisted continuous evolution to obtain a high-performance variant (evoBxb1). This evolved enzyme showed stronger DNA binding and catalytic activity in the complex, chromatin-rich environment of the nucleus, resulting in efficient DNA integration within its host. For application in *Bifidobacterium*, similar evolutionary strategies could be employed to improve recombinase stability and activity, such as reducing sensitivity to DNA methylation or host-specific inhibition. This would allow more efficient and reliable large-fragment integration in a genetically recalcitrant host like *Bifidobacterium*. For both CAST and LSR systems, optimizing delivery, expression, and activity specifically for *Bifidobacterium* will be critical. If the challenges described above can be addressed, it would enable stable integration of large genetic constructs, opening a major advancement in the genome editing field.

### Community editing

The ability to edit a bacterial genome within a community context offers new opportunities for both scientific discovery and expanded application in microbiome engineering to manipulate both the composition and the function of bacterial populations *in situ*. Specifically, editing *Bifidobacterium* within the gut can provide valuable insights into its role within the microbiome community. Additionally, editing an isolated strain outside the gut and then reintroducing it into the microbiome has limitations, as this approach can disrupt microbial composition and lead to unintended consequences. Developing an *in situ* editing strategy, however, allows for precise and controlled modifications within the microbiome, minimizing disruptions while enabling targeted genetic modifications.

The major challenges in community editing are efficient DNA delivery and targeted mutagenesis. While no studies have been performed in *Bifidobacterium* to date, efforts have been made to develop editing tools for the microbiome context. In a CAST DART (DNA-editing all-in-one RNA-guided CRISPR-Cas transposase) system, a single conjugative suicide CAST vector was developed that carries both the transposase gene and gRNA to direct the transposon insertion into a specific genome site of interest (86). This system enabled precise species and locus-specific edits within complex communities and has introduced targeted modifications in soils and infant gut microbiota *ex vivo*.

However, the conjugation-based delivery method does not allow species-specific delivery. Phage-based delivery, which uses the natural host specificity of bacteriophages, has been proposed as one possible solution to enhance the specificity of DNA delivery (87). One study demonstrated *in situ* editing in the soil microbiome by engineered λ phage to deliver CBE to *E. coli* and perform site-specific edits (88). Building upon this study, a more recent study was able to successfully utilize phage-derived particles to deliver base editors to bacteria within the gut of living mice (89). Here, the λ tail protein was engineered to enhance the adsorption of the phage to *E. coli,* enabling sufficient delivery of DNA. Additionally, a non-replicative DNA cosmid that does not replicate in the recipient bacteria was developed to prevent the spread of transgenes. These enhancements allowed the successful introduction of CBE into *E. coli* and *Klebsiella pneumoniae* within the gut environment. Although this study currently demonstrates base editing, the advancement of phage editing tools and the high capacity of λ phage present a new opportunity for a wider range of genetic modifications (90). Expanding on this opportunity, the more recently developed λ-DART system uses engineered λ phage particles to deliver a CAST transposase to enable targeted transposon insertions in complex microbial communities (91). This integration of a CRISPR-based system with transposases shows how phage-derived delivery platforms can enable precise genetic alterations and could be expanded with additional tools, such as recombinases, for greater versatility. Despite the abundance of prophages, a limited number of studies have investigated and characterized phages of bifidobacteria *in vitro* (92). The characterization and engineering of phages, along with advancements in *in situ* editing technologies, could enable precise genetic modifications of *Bifidobacterium* within the gut while preserving microbiome stability, opening new possibilities for functional studies and therapeutic applications. Given the pace and scale at which CRISPR, synthetic biology, and AI tools are evolving, we speculate that the next few years will substantially expand the available toolbox.

## CONCLUSION AND FUTURE PERSPECTIVES

The utilization of CRISPR-Cas system-based engineering tools in *Bifidobacterium* is still in its infancy. Common *Bifidobacterium* species and strains found in the human gut and used as probiotics encompass *B. lactis, B. breve, B. infantis, B. longum,* and *B. bifidum* strains (93). Considering the strain variability and the abundance of CRISPR-Cas systems across these species, there is a need for more extensive characterization and development of endogenous CRISPR-Cas-based editing tools. In parallel, advancing exogenous CRISPR-Cas-based editing tools will require identifying novel Cas effectors from closely related species and structure-guided mutagenesis to enhance their editing efficiency (94). Such advances will also lay the groundwork for expanding the CRISPR-based genome editing toolbox, including CAST, prime editing, and multiplex editing. Together, these efforts will provide new opportunities for in-depth study of *Bifidobacterium,* including functional genomics, probiotic strain engineering, and expanded therapeutic applications.

To date, the knockouts generated with CRISPR-based genome editing tools have been used as a proof of concept to demonstrate the potential of the toolbox. Future research can utilize these tools to generate knockouts to characterize the functions of genes involved in important metabolic and host interaction pathways. For instance, as one of the early colonizers of the infant gut, *B. infantis* is associated with beneficial effects such as modulation of weight and inflammatory markers (95). These effects are thought to be driven by its ability to consume host-derived glycans like human milk oligosaccharides (HMOs). Previous knockout studies using single and double crossover recombination have expanded our understanding of HMO metabolism in *B. longum* subsp. *longum* and *B. infantis,* identifying key fucosyllactose uptake systems and cross-feeding interactions (96, 97). Although core HMO utilization pathways have been mapped in a few model species, many strain- and substrate-specific functions remain less fully defined (98–101). With the aforementioned CRISPR-based tools that offer enhanced precision, efficiency, and specificity, future knockout studies can help validate the less-characterized pathways and the roles of low-prevalence genes in HMO metabolism across different *Bifidobacterium* strains.

Beyond genome editing, CRISPR interference/activation (CRISPRi/CRISPRa), a transcriptional regulation system in which deactivated Cas effectors are tethered to transcriptional repressors or activators, can be used to manipulate the expression of specific genes of interest. A study demonstrated this approach in *Streptomyces* by repurposing its endogenous Type I-E system by expressing the Cascade without Cas3 (102). In the same study, gene activation was also accomplished by fusing an activation domain to CasA, allowing recruitment of RNA polymerase to the promoter region. dCas12a-based CRISPRi has already been demonstrated in *B*. *catenulatum*, which was able to show repression of the *bcat* gene, resulting in decreased levels of branched-chain fatty acid production (78). More recently, a broadly applicable single plasmid CRISPRi system was developed, demonstrating efficient gene repression across multiple species, including *B. breve*, *B. animalis* subsp. *animalis*, *B. infantis*, *B. longum* subsp. *longum*, and *B. pseudocatenulatum* (103). A similar approach can be applied to *Bifidobacterium,* which will enable functional and regulatory analysis without generating full knockouts, making it useful for studying essential genes.

In addition to transcriptomic control, CRISPR-Cas-based tools can be leveraged to explore and reshape the epigenetic landscape (104). As shown previously, knockout of the restriction endonuclease enhances the uptake of foreign DNA (46). However, the R-M system has applications beyond foreign DNA recognition, as it is known to be relevant to functions such as cell division, DNA repair, and pili formation, enabling the cell to react to stressors (60). By utilizing synthetic epigenetic tools, such as CRISPRi/CRISPRa or CRISPR-guided demethylation, we can modulate these processes reversibly and precisely, expanding the application of *Bifidobacterium* as a probiotic (105).

After unraveling important metabolic pathways and relevant gene functions, the long-term objective would be to leverage the CRISPR-based editing tools to engineer strains for enhanced functionality or optimized performance. For example, cell wall components such as exopolysaccharides, peptidoglycans, and lipoteichoic acids are known to be relevant to immune modulation as they interact with immune receptors (106, 107). However, not only is there a limited understanding of their physiological properties, but the lack of editing tools has also prevented the modification of these cell wall components. After enhanced understanding of the biochemical attributes of these components, precise editing can optimize the length and composition of these polymers to achieve the most desirable phenotypes.

Continued development of CRISPR-Cas-based technologies will be important to fully unlock *Bifidobacterium* strains as genetically enhanced probiotics. Future efforts should focus not only on improving transformation efficiency and overcoming barriers associated with *Bifidobacterium* but also on expanding available tools beyond deletion, for precise insertions, gene regulation, and *in situ* modifications. Notably, the development of AI tools is not only making CRISPR-based editing more accessible by assisting from design to downstream analysis but also enabling the generation of novel Cas effectors (108, 109). Although still in their infancy, these approaches may later be applicable to a wider range of species, helping lower the barrier to CRISPR-based editing in diverse *Bifidobacterium*. These advancements will support functional studies and enable broader applications in microbiome research and therapeutic development.
